# Critical molecular pathways in CLL therapy

**DOI:** 10.1186/s10020-018-0001-1

**Published:** 2018-03-15

**Authors:** Gerardo Ferrer, Emili Montserrat

**Affiliations:** 1 Karches Center for Oncology Research, The Feinstein Institute for Medical Research, Northwell Health, Manhasset, NY USA; 2 Department of Hematology, Institute of Hematology and Oncology, University of Barcelona, Hospital Clínic, Villarroel 170, 08036 Barcelona, Spain

**Keywords:** CLL, BTK, PI3K, Bcl-2 and pathway inhibitors

## Abstract

Chronic lymphocytic leukemia (CLL), the most frequent type of leukemia in western countries, is characterized by the progressive accumulation in blood, bone marrow and lymphoid tissues of monoclonal B lymphocytes with a characteristic immunophenotype. Despite advances in therapy and improved outcome, in most instances CLL is an incurable disorder. Signaling via the B-cell receptor (BCR), the upregulation of anti-apoptotic proteins, and the cross-talk between CLL cells and microenvironment constitute key factors in the pathogenesis of CLL. Currently, inhibitors of kinases like BTK or PI3K blocking BCR signaling, and molecules that mimic the BH3 domain to compete with BCL-2 are established tools in the treatment of CLL. As the complex biology of CLL is rapidly unfolding, the number of small molecules targeting CLL molecular pathways is increasing and it is likely that they will further improve the outcome of patients with this form of leukemia.

## Background

Chronic lymphocytic leukemia (CLL) is the most frequent type of leukemia in western countries, with an incidence of 5.82/100000 inhabitants in the USA (Li et al., 2015). The median age at diagnosis is 72 years, with a higher incidence in males (1.7:1) (Li et al., 2015; Hallek, 2017; Kipps et al., 2017). CLL is characterized by the clonal expansion of B cells with a characteristic immunophenotype (i.e., smIg^weak^, CD29^+^, CD23^+^, CD20^weak^) that slowly accumulate in peripheral blood, bone marrow, and lymphoid tissues mainly as a result of defects in the apoptosis machinery such as the overexpression of Bcl2 family anti-apoptotic proteins (Hallek, 2017; Kipps et al., 2017; Billard, 2014). The clinical course of patients with CLL is heterogeneous with some patients succumbing a few months after diagnosis and others having prolonged survival and dying for reasons other than leukemia. The clinical heterogeneity of CLL does reflect differences in the biology of the disease, particularly the IGHV mutational status and chromosomal alterations (i.e., del13q, del11q, trisomy 12 and del17p). Beside del17p/TP53 mutation which is the strongest CLL biomarker for response to therapy, other mutations (e.g., *SF3B1, ATM, NOTCH1, BRIC3)* have been reported to correlate with the outcome of the disease, but they are not actionable yet (Lazarian et al., 2017).

In spite of major advances in its therapy, CLL remains largely incurable. However, in the past two decades important progress has been made in the understanding of the biology of CLL at different levels (e.g., B-cell receptor (BCR) signaling, anti- and pro-apoptotic proteins, and the microenvironment) (Kipps et al., 2017). This progress has resulted in the advent of small molecules and kinase inhibitors as new and effective therapies for CLL. In this paper, we review the main CLL molecular pathways that promote the survival and growth of CLL B cells and outline treatment results with agents targeting such pathways.

### B cell receptor and signaling

The BCR is a key for the fate of B cells and plays an important role in the survival of CLL cells. The BCR is connected to a network of kinases and phosphatases that tune and amplify its activation (Stevenson et al., 2011; Seda & Mraz, 2015). Each normal mature B cell has a unique antigen-binding site as a result of random rearrangement of IGH and IGL gene segments. The probability that two independent B cell clones present the same BCR is extremely small (10^− 9^ - 10^− 12^). However, approximately 30% of patients with CLL express similar, if not identical, BCRs with common (“stereotyped”) features (Stamatopoulos et al., 2017). This suggests that these cells are selected based on the structure of the antigen-binding domains of the smIg, presumably due to the recognition of similar antigens which could be associated with CLL pathogenesis. Patients within a BCR-stereotyped subset have a similar clinical behavior. Another important observation in the relationship between BCR and the mutational status of the immunoglobulin heavy chain variable region gene(*IGHV*). Patients with fewer than 2% of mutations present a more polyreactive BCR, poor biomarkers, more aggressive disease and shorter survival (Hamblin et al., 1999; Damle et al., 1999).

Continuous BCR signaling translates into the phosphorylation of intermediate members and secondary pathways such as AKT, ERK and NF-kB (Stevenson et al., 2011). Opposite to what is observed in diffuse large B cell lymphoma, this “tonic” signaling is not due to mutations in BCR signaling components (Davis et al., 2010; Philippen et al., 2010) but to the recognition of various autoantigens and other microbial or environmental components, including DNA, cytoskeletal non-muscle myosin heavy chain IIA, apoptotic cells, and LPS (Burger & Chiorazzi, 2013). In addition, CLL BCRs are able to bind each other and induce signaling; this is a peculiar feature only additionally observed in some murine polyreactive B1 cell BCRs (Duhren-von Minden et al., 2012). More recently, the crystal structures of a set of CLL BCRs led to the identification of the regions involved in the binding. Interestingly, the strength of the interaction seems to control the pace of the disease (Minici et al., 2017).

**Fig. 1:**
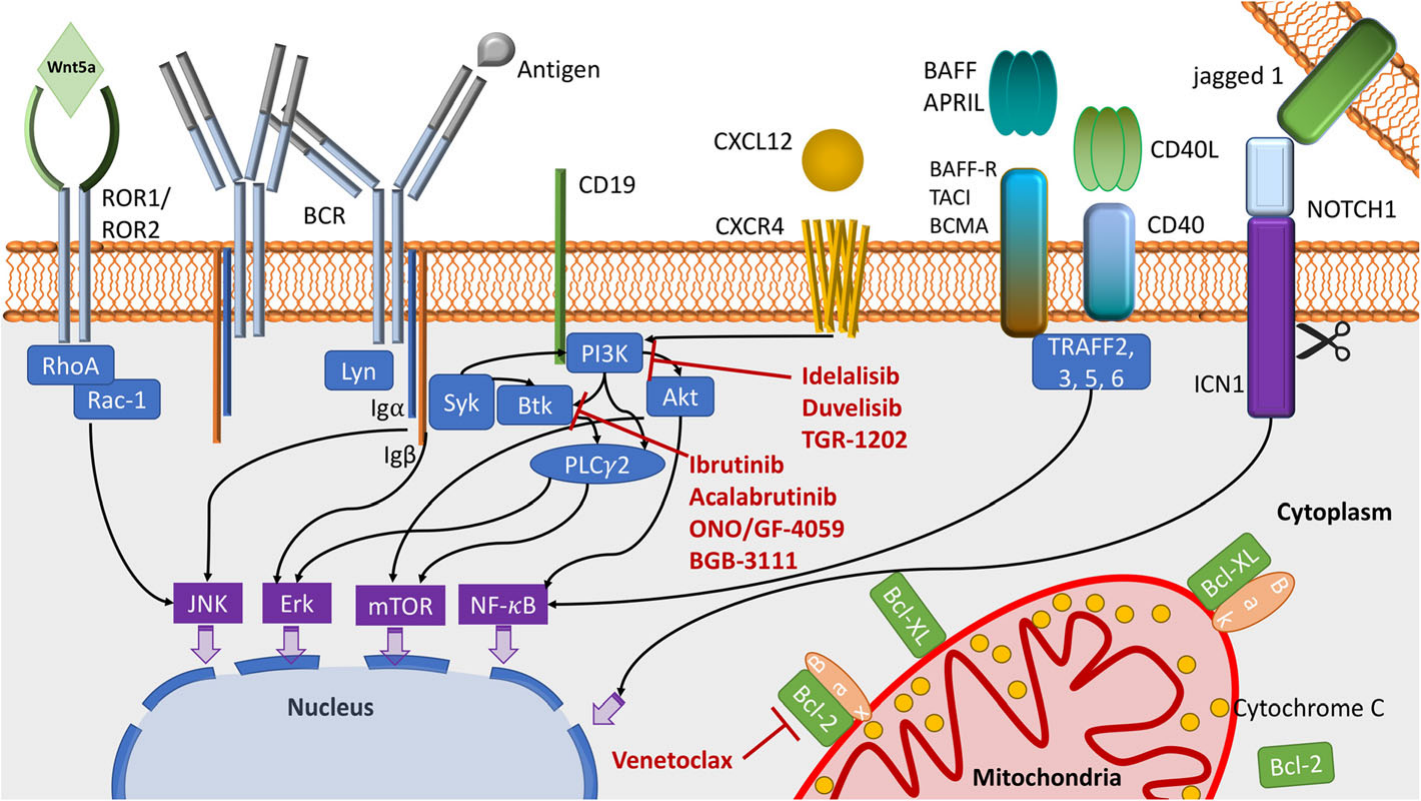
CLL main pathogenic pathways and target agents against BTK, PI3K and Bcl-2. BCR signaling is induced by the recognition of an antigen or by self-binding, Lyn promotes the phosphorylation of Iga and Igb that activates the spleen tyrosine kinase (Syk). Syk then triggers the formation of a multi-component ‘signalosome’, including Btk, Akt, PI3K and PLCγ2 among others. BCR co-receptor CD19 is important for PI3K activation, which recruits and activates PLCγ2, BTK and AKT. These leads to the activation of the c-Jun N-terminal kinase (JNK), MEK–extracellular signal-regulated kinase (ERK), mechanistic target of rapamycin (mTOR) and (NF-κB) signaling pathways. In addition, CLL cells activate these and other prosurvial, activatory pathways by their interaction with many soluble and surface factors. As an example: Wnt5a interact with the ROR1/ROR2 dimers promoting the activation of RhoA and Rac-1. CXCR4/CXCL12 engagement activates PI3K and downstream pathways, in addition other molecules. The TNF receptors CD40, BAFF-R, TACI and BCMA interact with their ligands CD40L or BAFF and APRIL, inducing the activation of the canonical and alternative NF-κB pathways depending on the TNF receptor-associated factor (TRAF). NOTCH1 signaling is initiated by the binding with one of the five ligands (e.g. jagged 1, Delta-like ligand 1 (DLL1)), followed by the release of the intracellular active portion (ICN1), enabling its migration into the nucleus. These pathways lead to the upregulation of anti-apoptotic molecules like Bcl-2, Bcl-XL and Mcl-1, sequestering the pro-apoptotic molecules Bax and Bak, and inhibiting the intrinsic apoptosis pathway. Inhibitors for PI3K, BTK and Bcl-2 are indicated in red

Finally, BCR binding initiates signaling by the phosphorylation of Igα and Igβ by Lyn and other Src family kinases. Then SYK, BTK, and PI3Ks turn active, modulating several pathways affecting survival, proliferation and migration of CLL cells (Fig. 1).

### Targets and agents blocking B-cell receptor signaling

#### Bruton’s tyrosine kinase, BTK

The gene encoding *BTK* is located on the X chromosome and was initially discovered in X-linked agammaglobulinemia (Vetrie et al., 1993). *BTK* is expressed in cells of all hematopoietic lineages except T- and plasma cells (Smith et al., 1994). It transmits, diversifies, and amplifies signals from a wide variety of surface molecules. BTK inhibitors block BCR signaling, thus strongly influencing cell survival, proliferation and migration (Burger & Buggy, 2013). In addition, BTK is critical to B cell motility and tissue homing, regulating the response to CXCL12 and integrins released by microenvironment cells (de Gorter et al., 2007), which explain the traffic of lymphocytes between lymph nodes and blood, and the lymphocytosis observed in subjects treated with BTK inhibitors (Chen et al., 2016; Woyach et al., 2014).

#### Ibrutinib

Ibrutinib is the first orally bioavailable small-molecule to inhibit BTK. It forms a covalent bond with Cys481 in the active site of BTK, preventing its phosphorylation (Honigberg et al., 2010). Despite a short circulating half-life, the covalent binding allows almost the complete occupancy of BTK active sites in peripheral blood mononuclear cells for 24 h (Advani et al., 2013). The first results of ibrutinib as single agent in the treatment of CLL were remarkable. The overall response rate (ORR) in previously treated patients with high-risk disease was 71%, and the 2-year progression free survival (PFS) was 75% (Byrd et al., 2013). These results were validated in consecutive trials. Of note, ibrutinib improved the outcome of patients with del17p and relapsed/refractory (R/R) disease with a median progression free survival approximating 30 months (Table 1) (Burger et al., 2014; Byrd et al., 2014; O'Brien et al., 2016). In a study with long follow-up (PCYC-1102/1103, NCT01105247/NCT01109069), 5- year PFS rates were 43% in 101 patients with R/R CLL and 92% in 31 treatment naïve (O'Brien et al., 2016). However, there is no plateau in the remission duration curves, and almost one third of patients discontinued treatment due to toxicity, CLL progression or Richter’s transformation (Woyach, 2017).

**Table 1:** Clinical trials with pathway inhibitors in CLL

Agent	N	Age in years	Median follow-up in months (Range)	TP53/	CK	ORR	PFS (2y)	OS (2y)	Ref.
(Study)	(R/R-TN)	(Range)		d17p		(CR)	(median in months)	(median in months)	
Ibrutinib	132	68	46	34	41	86%	n.r.	n.r.	(O'Brien et al., 2016)
(NCT01105247/NCT01109069)	(101-31)	(37-84)	(0-67)			(14%)	(not reached)	(not reached)	
Ibrutinib + rituximab	40	65	47	21	15/28	95%	62%	78%	(Jain et al., 2017)
	(36-4)	(35-82)	(36-51)			(23%)	(45mo)	(not reached)	
Ibrutinib	84	n.r.	34	53	n.r.	n.r.	84% at 3y	n.r.	(Ahn et al., 2017)
(NCT01500733)	(32-52)		(0.1-50)				(not reach)		
Ibrutinib	144	64	28	144	n.r.	83%	63%	75%	(O'Brien et al., 2016)
(NCT01744691)	(144-0)	(n.r.)	(n.r.)			(n.r.)	(not reached)	(not reached)	
Ibrutinib +/- rituximab +/- bendamustine	88	66	28	34/40	21/56	94%	n.a.	n.r.	(Thompson et al., 2015)
	(88-0)	(35-83)	(14-48)			(17%)			
Ibrutinib	195	67	19	79/154	39/153	90%	74%	86% at 1.5y	(Brown et al., 2018)
(NCT01578707)	(195-0)	(30-86)	(n.r.-26)				(n.r.)	(n.r.)	
Ibrutinib	621	60	17	26%	73/216	n.r.	64%	79%	(Mato et al., 2016)
Connect® CLL Registry	(536-80)	(22-95)	(n.r.)				(35mo)	(not reached)	
Ibrutinib	315	69	16	90/263	n.a.	n.r.	74% at 1y	84% at 1y	(Forum, 2016)
UK CLL Forum	(315-0)	(42-93)	(n.r.)				(n.r.)	(n.r.)	
Ibrutinib	95	69	10	50/80	n.a.	84%	75% at 1y	82% at 1y	(Winqvist et al., 2016)
Swedish CUP	(94-1)	(42-86)	(n.r.)			(3%)	(not reached)	(not reached)	
Idelalisib	54	63	n.r.	13	n.a.	72%	n.r.	n.r.	(Brown et al., 2014)
(NCT00710528)	(54-0)	(37-82)					(16mo)	(not reach)	
Idelalisib + rituximab	110	71	13	46	n.a.	81%	38%	72%	(Furman et al., 2014)
(NCT01539512)	(110-0)	(48-90)	(n.r.)			(0%)	(19mo)	(not reached)	
Idelalisib + ofatumumab	174	68	16	70	n.a.	75%	≈30%	≈65%	(Jones et al., 2017)
(NCT01659021)	(174-0)	(61-74)	(n.r.)			(<1%)	(16mo)	(not reached)	
Idelalisib + bendamustine	207	62	14	69	n.a.	n.r.	48%	75%	(Zelenetz et al., 2017)
(NCT01569295)	(207-0)	(56-69)	(n.r.)				(21mo)	(not reached)	
Venetoclax	116	66	17	31	n.a.	79%	52%	84%	(Roberts et al., 2016)
(NCT01328626)	(116-0)	(36-86)	(1-26)			(20%)	(25 mo)	(not reached)	
Venetoclax + rituximab	49	68	28	10/32	n.a.	86%	82%	94%	(Seymour et al., 2017)
(NCT01682616)	(49-0)	(50-88)	(1-42)			(51%)	(not reached)	(not reached)	
Venetoclax	107	67	12	107	n.a.	79%	72% at 1y	87% at 1y	(Stilgenbauer et al., 2016)
(NCT01889186)	(107-0)	(37-85)	(n.r.)			(16%)	(not reached)	(not reached)	

*R/R* Relapsed/Refractory, *TN* Treatment Naive, *CK* Complex Karyotype, *n.r.* No reported, *n.a.* No applicable, *mo* Months, *y* Year

CLL relapse on ibrutinib is mainly due to the acquisition of mutations in BTK or PLCG2, its direct downstream target (Woyach, 2017; Maddocks et al., 2015; Woyach et al., 2014). These mutations are found in 85–90% of patients at relapse by next generation sequencing (Ahn et al., 2017) and can be present a long time before clinical relapse is observed (Burger et al., 2016). The most common mutation in BTK is C481S, this mutation reduces the affinity of the ibrutinib-BTK binding, inducing a reversible inhibition (Woyach et al., 2014; Cheng et al., 2015). Conversely, PLCG2 mutations are associated with a gain-of-function, allowing signaling even when BTK is blocked (Woyach et al., 2014; Zhou et al., 2012).

The most important adverse events occurring with ibrutinib treatment are bleeding, hypertension, atrial fibrillation, cutaneous rash, and infections. Many of these side effects have been attributed to off-target effects of ibrutinib on epidermal derived growth factor receptor (EGFR), TEC family proteins, and ITK (interleukin-2–inducible tyrosine kinase). These side effects could be partially offset by second-generation BTK inhibitors in advanced clinical development such as ACP-196 (acalabrutinib), ONO/GS-4059, and BGB-3111, which virtually have no inhibitory effect over EGFR, TEC, and ITK (Wu et al., 2016). Inhibition of EGFR signaling has been associated with cutaneous rash and severe diarrhea (Lynch Jr. et al., 2007), while the risk of bleeding might be due to the dependency of platelets to TEC for platelet aggregation (Atkinson et al., 2003; Hamazaki et al., 1998). Finally, a set of T cells are dependent on TEC and ITK signaling (Andreotti et al., 2010), thus new generation BTK inhibitors might improve anti-tumoral T cell responses.

#### PI3K

Phosphoinositide-3 kinase (PI3K) was initially described in association with the polyoma middle T protein (Whitman et al., 1985). PI3Ks affect a diverse array of biological processes in cells, including proliferation, differentiation, survival, and metabolism. BCR activation induces the recruitment of CD19 along with PI3K to the membrane to propagate and amplify signaling (Fig. 1). PI3K class 1 is constituted by a catalytic and a regulatory subunit, and each subunit has different isoforms. The catalytic subunits include α, β, γ and δ isoforms and the regulatory subunit involves p85 and p101/55. Importantly, the catalytic subunits α and β are expressed ubiquitously, while the subunits γ and δ are present predominantly in leukocytes. It is considered, therefore, that the inhibition of these subunits could reduce treatment toxicity (Engelman et al., 2006; Vanhaesebroeck et al., 2010; Furman et al., 2014).

#### Idelalisib

Idelalisib (CAL-101, GS-1101) is the first orally competitive inhibitor of the PI3Kδ isoform, able to block survival and homing signals in CLL including those produced by the BCR and the chemokine receptors CXCR4 and CXCR5 (Brown et al., 2014; Lannutti et al., 2011). In a phase I study in patients with relapsed CLL, idelalisib resulted in an ORR of 72% and PFS at 16 months of 50% (Brown et al., 2014). Subsequent phase III trials investigated idelalisib in combination with rituximab or ofatumumab resulting in a significant increase of the PFS and OS as compared to idelalisib alone (Furman et al., 2014; Jones et al., 2017). However, these responses were shorter than in patients treated with ibrutinib (Table 1). Interestingly, TP53 lesions and complex karyotype do not seem to reduce treatment effectiveness, but the number of trials is small and their follow-up short (Fruman & Cantley, 2014; Kreuzer et al., 2016) suggesting caution in drawing robust conclusions about this otherwise important issue.

Patients treated with idelalisib present an unusual pattern of toxicity including enteritis/diarrhea (< 20%) (not infrequently appearing as “late” events), transaminitis (< 15%) and pneumonitis (< 5%). Fifteen percent of patients present a grade 3–4 toxicities and are generally responsible for idelalisib discontinuation (Lampson et al., 2016; Louie et al., 2015). Importantly, in treatment-naïve patients the combination of idelalisib with anti-CD20 monoclonal antibodies may result in severe liver and gastrointestinal toxicity mediated by CD8^+^ T cells (Lampson et al., 2016; Louie et al., 2015). Due to opportunistic infections, monitoring CMV and prophylaxis for *Pneumocystis jirovecii* pneumonia is recommended (Cheah & Fowler, 2016).

Two other PI3K inhibitors are in advanced clinical development. Duvelisib is a dual PI3K δ and γ inhibitor, and TGR-1202 is a next-generation δ inhibitor with a reduced hepatic toxicity and colitis compared to other PI3Kδ inhibitors (Balakrishnan et al., 2015; Mato et al., 2016).

### Apoptosis

Apoptosis or programmed cell death is a critical process for the development, homeostasis and the prevention of tumorigenesis. Escaping the apoptotic program is one of the hallmarks of cancer and a key mechanism in resistance to therapy (Hanahan & Weinberg, 2011). The apoptotic program is constituted by two main activation pathways: the extrinsic, initiated by the ligation of death receptors, and the intrinsic, where several intracellular signals control the balance between anti-apoptotic Bcl-2 family members and the pro-apoptotic Bax and Bak to initiate the mitochondrial membrane permeabilization (Fig. 1). These two pathways converge in activating the caspase family of proteases (Strasser et al., 2011).

The prolonged survival of CLL cells is in part associated with defective apoptosis and microenvironment mediated signals (e.g., CD40L, BAFF, APRIL, CXCL12, and VACM-1 (Kipps et al., 2017; ten Hacken & Burger, 2014) that trigger the NF-KB and PI3K/AKT pathways. These pathways are constitutively activated in CLL and promote the overexpression of Bcl-2 family members. In fact, if Bcl-2 and Mcl-1 are suppressed, CLL cells undergo apoptosis (Hussain et al., 2007).

Approximately half of all patients with CLL have a leukemic clone with a deletion on 13q which includes the loss of two microRNAs miR-15a and miR-16-1. These microRNAs diminish the expression of several proteins including the anti-apoptotic Bcl-2 (Cimmino et al., 2005). Another common alteration in CLL cells is the mutation of *TP53* or the deletion of 17p that contains this gene, an important apoptosis regulator (Zenz et al., 2009; Pospisilova et al., 2012; Bieging et al., 2014).

### Targets and agents regulating CLL apoptosis

#### BCL-2

The B-cell lymphoma 2 (*BCL-2)* gene is located in the chromosome 18 and was initially discovered in the chromosomal translocation t(14;18)(q32;q21) in follicular lymphomas (Tsujimoto et al., 1984). BCL-2 locates at the outer membrane of the mitochondria, sequestering the proapoptotic proteins Bax and Bak and preventing them from oligomerization, and inducing mitochondrial membrane permeabilization. The sequestered Bax and Bak can be released by family’s BH3-only members such as Bim, Puma, Bid, Bad, and Noxa by binding to and antagonizing Bcl-2 family proteins (Strasser et al., 2011). Bcl-2 has been found to be overexpressed in CLL cells, where it mediates tumor cell survival and has been associated with resistance to therapy (Billard, 2012; Shehata et al., 2010).

#### Venetoclax

Venetoclax is an oral small-molecule designed to block Bcl-2 prosurvival activity by emulating the BH3 domain. Venetoclax is a re-engineering of navitoclax, which showed clinical efficacy but was accompanied with thrombocytopenia caused by Bcl-XL inhibition (Souers et al., 2013). Venetoclax was first evaluated in 3 refractory patients with CLL and resulted in tumor lysis syndrome in 24 h (Souers et al., 2013). Tumor lysis syndrome is due to rapid leukemic cell death and the release of their cellular contents into the blood stream. Consequently, a dose escalation schedule was chosen in a phase II trial on refractory del(17q) patients with CLL (Stilgenbauer et al., 2016). With a median follow-up slightly longer than 12 months, the ORR was 85% and the CR rate was 8%. Of note, the ORR is not superior to that found with BTK or PI3K inhibitors, but a proportion of patients achieve CR with undetectable MRD. In CLL, venetoclax showed to be well tolerated but some patients presented grade 3–4 adverse effects including neutropenia, infections, anemia and thrombocytopenia. These results suggest that Bcl-2 inhibition induced by venetoclax offers an opportunity to effectively treat patients with del (17p) (Table 1). Resistance to venetoclax is largely driven by microenvironment signals that promote the expression of Mcl-1 and Bcl-XL, reducing venetoclax efficiency, especially in nodal tissues (Bose et al., 2017). Notably, therapy with BCR axis inhibitors such as ibrutinib (Cervantes-Gomez et al., 2015), acalabrutinib (Patel et al., 2017a), and duvelisib (Patel et al., 2017b) results in a decline in MCL-1 protein levels in CLL cells, providing a mechanism-based rationale to combine them with venetoclax. Such treatment combinations (e.g. venetoclax + ibrutinib) are currently under investigation (Billard, 2014; Bose et al., 2017; Seymour et al., 2017).

### Microenvironment

Neoplastic CLL cells are highly dependent on interactions with the microenvironment for their survival and proliferation. Main components of the microenvironment are monocyte-derived nurselike cells (NLCs), and other myeloid cells, mesenchymal-stromal cells, T cells and NK cells, all of which communicate with CLL cells through a complex and intertwined network of adhesion molecules, chemokine receptors, tumor necrosis factor (TNF) family members, and soluble factors (reviewed in (Ten Hacken & Burger, 2016).

The CLL microenvironment is becoming an important treatment target. Lenalidomide is an orally active immunomodulatory agent, which interferes with a wide variety of the components of the CLL microenvironment. Lenalidomide does not induce apoptosis of leukemic cells in vitro, but in vivo it upregulates receptors of B cell activation on CLL cells, together with an enhancement of the host immune response, thus inducing the recognition of CLL cells (Chanan-Khan et al., 2011; Chen et al., 2011). The mode of action is disease specific but cereblon has been identified as the main target of lenalidomide and may underlie many of its effects (Itchaki & Brown, 2017). As a single agent, lenalidomide is effective in CLL R/R and treatment-naïve patients (Chen et al., 2011; Badoux et al., 2011; Ferrajoli et al., 2008). In addition, it has been studied in combination with rituximab and more recently with ibrutinib (Ferrajoli et al., 2008; Pollyea et al., 2014). Results of lenalidomide given as maintenance therapy are promising (Fink et al., 2017; Foà et al., 2016).

### CLL active pathways mediated by the microenvironment

#### WNT signaling

WNT was first described in a model of breast cancer (Nusse & Varmus, 1982). Canonical and non- canonical WNT signaling are both active in CLL; this is due to the high levels of several molecules produced by the microenvironment including Wnt3, Wnt5b, Wnt6, Wnt10a, Wnt14, and Wnt16 (Lu et al., 2004; Yu et al., 2016). In contrast to mature normal leukocytes, CLL cells express on their surface ROR1, a tyrosine-kinase-like transmembrane receptor whose higher expression level has been associated with a worse patient’s outcome (Cui et al., 2016). WNT5a crosslinks ROR1/ROR2 inducing the activation of RAC1 and RHOA and thereby promoting CLL cell proliferation and migration (Fig. 1) (Yu et al., 2016). An anti-ROR1 monoclonal antibody is currently being investigated in a phase 1 clinical trial (NCT02222688/NCT02860676) as a new treatment for CLL (Choi et al., 2015; Yu et al., 2017). In addition, mutations and methylation alterations in members of this pathway have been observed, together with the finding of a CLL risk locus at the *LEF1* gene (Kulis et al., 2012; Wang et al., 2014; Berndt et al., 2013).

#### NOTCH1 signaling

Notch signaling is an evolutionary conserved signaling system shared by many multicellular organisms (Artavanis-Tsakonas et al., 1999). In humans there are four receptors NOTCH1, NOTCH2, NOTCH3 and NOTCH4 (Andersson et al., 2011)*.* These transmembrane receptors bind to ligands that are other transmembrane proteins; thus, the signaling is induced by cells in proximity (Fig. 1). Receptor-ligand crosslinking promotes conformational changes, inducing the release of the intracellular portion to the nucleolus and provoking the expression of several genes (Kopan & Ilagan, 2009). Mutations of *NOTCH1* can be observed in 2%–14% of patients with CLL and have been correlated with resistance of anti-CD20 monoclonal antibodies, treatment refractoriness, and disease transformation (Fabbri et al., 2011; Puente et al., 2011; Quesada et al., 2011; Rossi et al., 2012; Wang et al., 2011). The majority of NOTCH1 mutations increase the stability of the intracellular portion (Fabbri & Dalla-Favera, 2016). In addition, NOTCH1 signaling is active in the leukemic cells in a high proportion of patients (50%) despite the absence of mutation. This is believed to be due to the expression of the ligands on the CLL cells and the microenvironment, but other ligand-independent mechanisms (e.g. cross activation by other signaling pathways, vesicle trafficking) could also participate (Fabbri et al., 2017; Rosati et al., 2009).

#### NF-kB

The NF-kB (nuclear factor kappa-light-chain-enhancer of activated B cells) as indicated by its name was first described in B cells (Sen & Baltimore, 1986). However, NF-kB is present in many cells and controls the expression of genes that influence the immune response, growth, differentiation, survival, development, as well as tumorigenesis and metastasis (Smale, 2011). Two NF-kB main activation pathways have been described: the classical or canonical pathway and alternative or non-canonical pathway (Hayden & Ghosh, 2008). In CLL, both signaling pathways seem to be activated by microenvironment-related molecules such as CD40L for the canonical pathway and BAFF or APRIL for both, the canonical and non-canonical pathway (Fig. 1) (Furman et al., 2000; Endo et al., 2007; Ferrer et al., 2014). Mutations affecting these pathways are infrequent in CLL; thus alterations in BIRC3 are observed in 0.4% of patients at diagnosis and 8.6% in patients requiring treatment (Baliakas et al., 2015; Chiaretti et al., 2014; Cortese et al., 2014). Mutations in *MYD88* can also induce the activation of the NF-kB. MyD88 is an adaptor protein required for signaling from Toll-like receptors and receptors of the interleukin-1 family. In CLL, approximately 3% of cases present mutations in this gene and most of them represent the gain-of-function L265P mutation that promotes the constitutive activation of the NF-kB (Puente et al., 2011; Landau et al., 2013; Mansouri et al., 2016; Ntoufa et al., 2016). Finally, NF-kB activation by BAFF in CLL cells can induce the activation of BCR signaling by SYK and avoid the BTK and PI3K inhibition (Paiva et al., 2017).

#### CXCR4/CXCL12 signaling

The chemokine stromal cell-derived factor-1 (SDF-1)/CXCL12 is the single ligand for the chemokine receptor CXCR4 (Fig. 1). The interactions between CXCL12 and CXCR4 affect the survival, proliferation, and migration of cells as well as angiogenesis and tumor metastasis. Both molecules are widely expressed by multiple cell types, including several immune cells, stem cells, endothelial cells and stromal cells (Guo et al., 2016; Han et al., 2014). In CLL, CXCR4 is highly expressed on the membrane of the peripheral blood leukemic cells to move into a better environment (Mohle et al., 1999). Chiorazzi’s group identified CXCR4 as a key molecule in their model of the life-cycle of CLL cells, where the surface expression of CXCR4 is associated with the location of the leukemic cells; leaving and entering the tissue (Calissano et al., 2011). Treatment strategies targeting both proteins are under investigation. CXCR4 antagonists include T140, AMD3100 (Plerixafor) and MDX-1338/BMS 93656 (Burger & Peled, 2009). In a phase I clinical trial in relapsed CLL patients, mobilization of CLL cells to the peripheral blood was observed with plerixafor in combination with rituximab (NCT00694590) (Andritsos et al., 2010). NOX-A12 is a Spiegelmer (L-RNA aptamer) that binds and antagonizes CXCL12. In vitro, it inhibits CLL-cell migration and sensitizes to cytotoxic agents (Hoellenriegel et al., 2014). NOX-A12 has been tested in relapsed CLL patients in combination with bendamustine and rituximab in a phase IIa trial (NCT01486797) (Steurer et al., 2014).

## Conclusions

The pace at which the understanding of the biology of CLL has increased over the last two decades is impressive and it has opened the door to new treatment compounds that target specific disease pathways (i.e. BCRi and BCL2i). These agents are effective in disease settings (e.g. del17p/TP53mutations) where conventional chemo(immuno)therapy fails. As a result, treatment algorithms for CLL are changing. Hopefully, as other critical molecular pathways and their interactions (e.g. ROR1, NOTCH1 BAFF/APRIL, CXCR4/CXCL12, PD1/PDL1, Mcl-1, SYK, CDK9 and CSF1R) are unraveled, newer and more effective pathway inhibitors will appear and so on hold promise for an even more effective therapeutic armamentarium. The introduction of CLL pathways inhibitors, however, does not convey the end of chemo(immuno) therapy in the management of this form of leukemia, rather their combined use is a most promising strategy in CLL therapy. Pathway inhibitors pose some issues that deserve investigation: best treatment combinations (e.g. BCRi + BCL2i, monoclonal antibodies [and or cytotoxic agents] + pathway inhibitors); optimal dose and schedule (including treatment duration), identification of specific predictive factors, mechanisms of resistance to therapy, and how to overcome them are the most important questions to be addressed.
